# Discovery and validation of dominantly inherited Alzheimer’s disease mutations in populations from Latin America

**DOI:** 10.1186/s13195-022-01052-1

**Published:** 2022-08-05

**Authors:** Leonel Tadao Takada, Carmen Aláez-Verson, Bhagyashri D. Burgute, Ricardo Nitrini, Ana Luisa Sosa, Raphael Machado Castilhos, Marcia Fagundes Chaves, Erika-Mariana Longoria, Karol Carrillo-Sánchez, Sonia Maria Dozzi Brucki, Luis Leonardo Flores-Lagunes, Carolina Molina, Marcos Jimenez Olivares, Ellen Ziegemeier, Jennifer Petranek, Alison M. Goate, Carlos Cruchaga, Alan E. Renton, Maria Victoria Fernández, Gregory S. Day, Eric McDade, Randall J. Bateman, Celeste M. Karch, Jorge J. Llibre-Guerra, for the Dominantly Inherited Alzheimer Network

**Affiliations:** 1 Department of Neurology, Hospital das Clinicas, University of São Paulo Medical School, São Paulo, Brazil; 2 Laboratorio de Diagnóstico Genómico, Instituto Nacional de Medicina Genómica, Ciudad de México, México; 3 Department of Psychiatry, Washington University School of Medicine, St Louis, MO USA; 4 Instituto Nacional de Neurología y Neurocirugía, Ciudad de Mexico, Mexico; 5 Neurology Service, Hospital de Clínicas de Porto Alegre, Porto Alegre, Brazil; 6 Department of Internal Medicine, Faculty of Medicine, Universidade Federal do Rio Grande do Sul, Porto Alegre, Brazil; 7 Department of Neurology, Washington University School of Medicine, St Louis, MO USA; 8 Department of Neuroscience, Icahn School of Medicine at Mount Sinai, New York, NY USA; 9 Department of Neurology, Mayo Clinic Florida, Jacksonville, FL USA

**Keywords:** Dominantly inherited Alzheimer disease, Presenilin 1, Latin America, Early-onset Alzheimer disease

## Abstract

**Background:**

In fewer than 1% of patients, AD is caused by autosomal dominant mutations in either the *presenilin 1* (*PSEN1*), *presenilin 2* (*PSEN2*), or *amyloid precursor protein* (*APP*) genes. The full extent of familial AD and frequency of these variants remains understudied in Latin American (LatAm) countries. Due to the rare nature of these variants, determining the pathogenicity of a novel variant in these genes can be challenging. Here, we use a systematic approach to assign the likelihood of pathogenicity in variants from densely affected families in Latin American populations.

**Methods:**

Clinical data was collected from LatAm families at risk for DIAD. Symptomatic family members were identified and assessed by local clinicians and referred for genetic counseling and testing. To determine the likelihood of pathogenicity among variants of unknown significance from LatAm populations, we report pedigree information, frequency in control populations, *in silico* predictions, and cell-based models of amyloid-beta ratios.

**Results:**

We identified five novel variants in the *presenilin1* (*PSEN1*) gene from Brazilian and Mexican families. The mean age at onset in newly identified families was 43.5 years (range 36–54). *PSEN1* p.Val103_Ser104delinsGly, p.Lys395Ile, p.Pro264Se, p.Ala275Thr, and p.Ile414Thr variants have not been reported in PubMed, ClinVar, and have not been reported in dominantly inherited AD (DIAD) families. We found that *PSEN1* p.Val103_Ser104delinsGly, p.Lys395Ile, p.Pro264Se, and p.Ala275Thr produce Aβ profiles consistent with known AD pathogenic mutations. *PSEN1* p.Ile414Thr did not alter Aβ in a manner consistent with a known pathogenic mutation.

**Conclusions:**

Our study provides further insights into the genetics of AD in LatAm. Based on our findings, including clinical presentation, imaging, genetic, segregations studies, and cell-based analysis, we propose that *PSEN1* p.Val103_Ser104delinsGly, p.Lys395Ile, p.Pro264Se, and p.Ala275Thr are likely pathogenic variants resulting in DIAD, whereas *PSEN1* p.Ile414Thr is likely a risk factor. This report is a step forward to improving the inclusion/engagement of LatAm families in research. Family discovery is of great relevance for the region, as new initiatives are underway to extend clinical trials and observational studies to families living with DIAD.

## Introduction

Alzheimer’s disease (AD) is the most common cause of dementia [1] and has emerged as an important societal issue and a global priority [2]. In the absence of clinically meaningful disease-modifying treatments, the number of adults with dementia worldwide has been projected to more than triple by 2050 relative to 2010 levels [3–5]. Much of the increase is projected to occur in low and middle-income countries (LMICs) [6]. Countries in Latin America (LatAm) will experience the largest dementia increase and impact. However, genetics of early-onset AD remain markedly under-explored in LatAm populations [7–9].

Genetic determinants of AD include pathogenic variants in the *amyloid precursor protein* (*APP*), *presenilin 1* (*PSEN1*), or *presenilin 2* (*PSEN2*) genes leading to dominantly inherited AD (DIAD) [10, 11], and susceptibility loci harboring alleles that modify the risk of developing the disease [12, 13]. Despite its low frequency relative to sporadic AD, families living with dominantly inherited Alzheimer disease (DIAD) mutations face a higher burden of disease due to the significant early onset.

To date, more than twenty-four dominantly inherited Alzheimer disease (DIAD) pathogenic variants have been reported in Latin American (LatAm) countries, including twenty-one *PSEN1*, two *PSEN2*, and one *APP* variant, with unique characteristics including the presence of common ancestors, evidence of a high grade of admixture and ancestry background (e.g., African, Western European, Asia, and Native American) and presence in large extended families following regional distribution usually related to founder effects [11]. Yet, the number and distribution of DIAD pathogenic variants in LatAm remain underestimated [9, 11, 14]. In 2019, the Dominantly Inherited Alzheimer Network (DIAN), an international network designed to follow families with mutations in *APP*, *PSEN1*, and *PSEN2* that cause DIAD, launched the DIAN-LatAm initiative to identify new DIAD families from LatAm countries and offer research opportunities and experimental therapies to prevent, delay or treat AD. The DIAN-LatAm network includes six performance sites in Mexico (Guadalajara and Mexico City), Colombia (Medellín), Brazil (Sao Paulo), and Argentina (Buenos Aires, and Salta). Since its inception, the DIAN-LatAm and DIAN Expanded Registry (DIAN EXR; https://dian.wustl.edu/our-research/registry/) have collaborated to identify new families with AD mutations and provide them information on research opportunities, creating a cohort-ready population that may accelerate study recruitment and completion. In a partnership between DIAN and LatAm countries (e.g., Mexico, Costa Rica, Brazil, Colombia, Chile, and Argentina, among others), we aimed to identify new pathogenic variants of familial Alzheimer’s in Latin America and refine the pathogenicity criteria. In this effort, we have screened 34 families at risk for DIAD (families with a history of early-onset dementia in two or more generations), identifying 15 families with known pathogenic variants [11] and five with variants of unknown significance. Two families were negative for DIAD variants or other genes associated with autosomal dominant cause of dementia (e.g., MAPT, GRN, TARDBP, FUS), probands on those families were *APOE4* carriers; 11 families are undergoing additional genetic counseling for future genetic testing. In this manuscript, we prioritize novel variant descriptions over newly-identified carriers of previously identified variants. Frequency, distribution by country, and clinical characteristics of known pathogenic variants have been described elsewhere [11].

Here, we describe five new families from LatAM (four from Brazil and one from Mexico) with multiple generations of early-onset AD (<65 years). At the time of enrollment, whether the previously uncharacterized variant is the cause of disease in these families remained unknown.

To address this gap, we took a multi-pronged approach to determine the pathogenicity of these five *PSEN1* variants by performing clinical-cognitive, genetic, and cell-based analyses.

## Methods

### Participant consent

Participant evaluation was done in accordance with The Code of Ethics of the World Medical Association (Declaration of Helsinki). All individuals signed an informed consent approved by the Institutional Review Board of the University of Sao Paulo, School of Medicine (Brazil), the National Institute of Neurology and Neurosurgery (Mexico), and Instituto Nacional de Medicina Genómica (Mexico).

### Clinical-cognitive assessments

Clinical data was collected from five LatAM families at risk for DIAD (two or more generations with a family history of early-onset dementia (age of onset <65). Symptomatic family members were identified and assessed by local clinicians and referred for genetic counseling and testing. At least one member per family (proband/index case) underwent medical, neurological, and neuropsychological evaluations, including the Mini-Mental State Examination (MMSE) [15] or Montreal Cognitive Assessment (MoCA) [16] with additional neuropsychological tests (e.g., verbal fluency, logical memory) and dementia functional scales (e.g., Clinical Dementia Rating (CDR®) [17], Functional Activities Questionnaire). Clinical diagnosis of symptomatic AD or cognitive status (cognitive healthy vs. cognitive impaired) was done without knowledge of mutation status and according to DSM-IV criteria.

### Genetic screening and counseling

The genetic counseling and testing followed the Huntington Disease Society of America’s Guidelines for Genetic Testing for Huntington Disease [18], which is considered the gold standard for genetic testing for adult-onset conditions. All families received two–three consultations, including pre-test sessions and post-test disclosure.

#### Isolation of genomic DNA

For the Brazilian families, DNA was isolated from buccal swab brushes. A DNA sample from at least one proband per family was sequenced via Mendelics® laboratory. Family segregation studies were performed in two out of five families. For the Mexican family, DNA was extracted using Maxwell® 16 Blood DNA Purification Kit (Promega, Madison, WI, USA) according to the manufacturer’s recommendations. The purity and concentration of the DNA sample were measured using NanoDrop 1000 spectrophotometer (Thermo Fisher Scientific, Waltham, MA, USA) and Qubit fluorometer (Thermo Fisher Scientific, Waltham, MA, USA).

#### Massive parallel sequencing (NGS)

Exome sequencing was performed with the CES v2 kit (Sophia Genetics DDM, Saint Sulpice, Switzerland). Library preparation was done according to the manufacturer’s protocol. The sequencing was performed on NextSeq 500 Instrument (illumina San Diego, CA). For mutation screening, we focused on missense, nonsense, splicing, and frameshift variants in Mendelian AD (*PSEN1*, *PSEN2*, and *APP*), frontotemporal dementia (*MAPT*, *GRN*, *TARDBP*, *VCP*, *CHMP2B*, *FUS*, and *TBK1*), and prion disease genes (*PRNP*). Variants not previously reported in (1) the Human Gene Mutation Database (HGMD) (www.hgmd.cf.ac.uk) or ClinVar (https://www.ncbi.nlm.nih.gov/clinvar/), (2) the AlzForum (www.alzforum.org/mutations) database, or (3) in a literature search, were considered as novel variants.

#### Bioinformatic analysis of novel variants

To determine the frequency of the novel variants in the population, we examined two population-based exome sequencing databases: the Exome Variant Server (EVS, https://evs.gs.washington.edu/EVS/) and the Genome Aggregation Database (GnomAD, https://gnomad.broadinstitute.org/). Sorting Intolerant From Tolerant (SIFT) [19] was used to predict whether the amino acid change would be disruptive to the encoded protein. The Combined Annotation Dependent Depletion (CADD, https://cadd.gs.washington.edu/score) score was used to bioinformatically predict the pathogenicity of the variants [20]. Final pathogenicity classification was assigned as described by Hsu et al. [21]. All variant nomenclature is based on the hg19 reference genome assembly and *PSEN1* transcript ENST00000324501.5.

### Biochemical analysis

#### Cloning and site-directed mutagenesis

The full-length *PSEN1* cDNA cloned into pcDNA3.1 myc/his vector was used for mutagenesis (Brunkan et al., 2005). *PSEN1* variants of unknown significance (Table 1) were introduced into the *PSEN1* cDNA using a QuikChange Lightning Site-Directed Mutagenesis Kit (Agilent Technologies, Santa Clara, CA, USA). Clones were sequenced to confirm the presence of the variant and the absence of additional modifications. *PSEN1* WT and the pathogenic *PSEN1* exon 9 deletion (ΔE9) mutation were included as controls.

**Table 1:** Clinical characteristics of identified variants

***PSEN1*** variants and number of family members	AAO mean (Range)	LATAM Country report	Predominant clinical presentation	Clinical domains with significant impairment throughout the disease				
				No. affected family members	Memory	Behavior	Motor	Language
p.Val103_Ser104delinsGly *n*=13	37.8 (34–38)	Brazil	Amnestic/language	7	+++	−	+	++
p.Ala275Thr *n*=4	50.5 (45–56)	Brazil	Amnestic/behavioral	2	+++	++	−	++
p.Lys395Ile *n*=7	51.7 (50–54)	Brazil	Amnestic	4	+++	+	++	−
p.Pro264Ser *n*=7	48.5 (45–52)	Brazil	Amnestic	3	+++	−	−	−
p.Ile414Thr *n*=12	54.0 (50–58)	Mexico	Amnestic/behavioral	6	++	++	+	−

*AAO* Age at onset

#### Transient transfection

Mouse neuroblastoma cells in which endogenous *PSEN1* and *PSEN2* were knocked out by CRISPR/Cas9 (N2A-PS1/PS2 KO) [22] were used to assess the impact of variants of unknown significance in *PSEN1* on Aβ levels. Human APP WT (695 isoform) was co-transfected along with the *PSEN1* constructs. N2A-PS1/PS2 KO cells were maintained in equal amounts of Dulbecco’s modified Eagle’s medium and Opti-MEM, supplemented with 5% fetal bovine serum, 2 mM L-glutamine, and 100 μg/mL penicillin/streptomycin. Upon reaching confluency, cells were transiently transfected with Lipofectamine 2000 (Life Technologies). Culture media was replaced after 24 h, and cells were incubated for another 24 h prior to analysis of extracellular Aβ in the media.

#### Aβ Enzyme-linked immunosorbent assay (ELISA)

Conditioned media was collected and centrifuged at 3000×g at 4°C for 10 min to remove cell debris. Levels of Aβ40 and Aβ42 in cell culture media were measured by sandwich ELISA as directed by the manufacturer (Life Technologies, Carlsbad, CA, USA). Statistical difference was measured using a one-way ANOVA and post hoc Dunnett test.

#### Immunoblotting

Cell pellets were extracted on ice in lysis buffer (50 mM Tris pH 7.6, 2 mM EDTA, 150 mM NaCl, 1% NP40, 0.5% Triton 100×, protease inhibitor cocktail) and centrifuged at 14,000×g at 4°C. Protein concentration was measured by BCA as described by the manufacturer (Pierce-Thermo). Standard SDS-PAGE was performed in 4–20% Criterion TGX gels (Bio-Rad). Samples were boiled for 5 min in Laemmli sample buffer prior to electrophoresis (Laemmli, 1970). Immunoblots were probed with 6E10 (1:1000; Millipore) and goat-anti-mouse-HRP (1:5000; Thermo Fisher).

### Pathogenicity algorithm

**Fig. 1:**
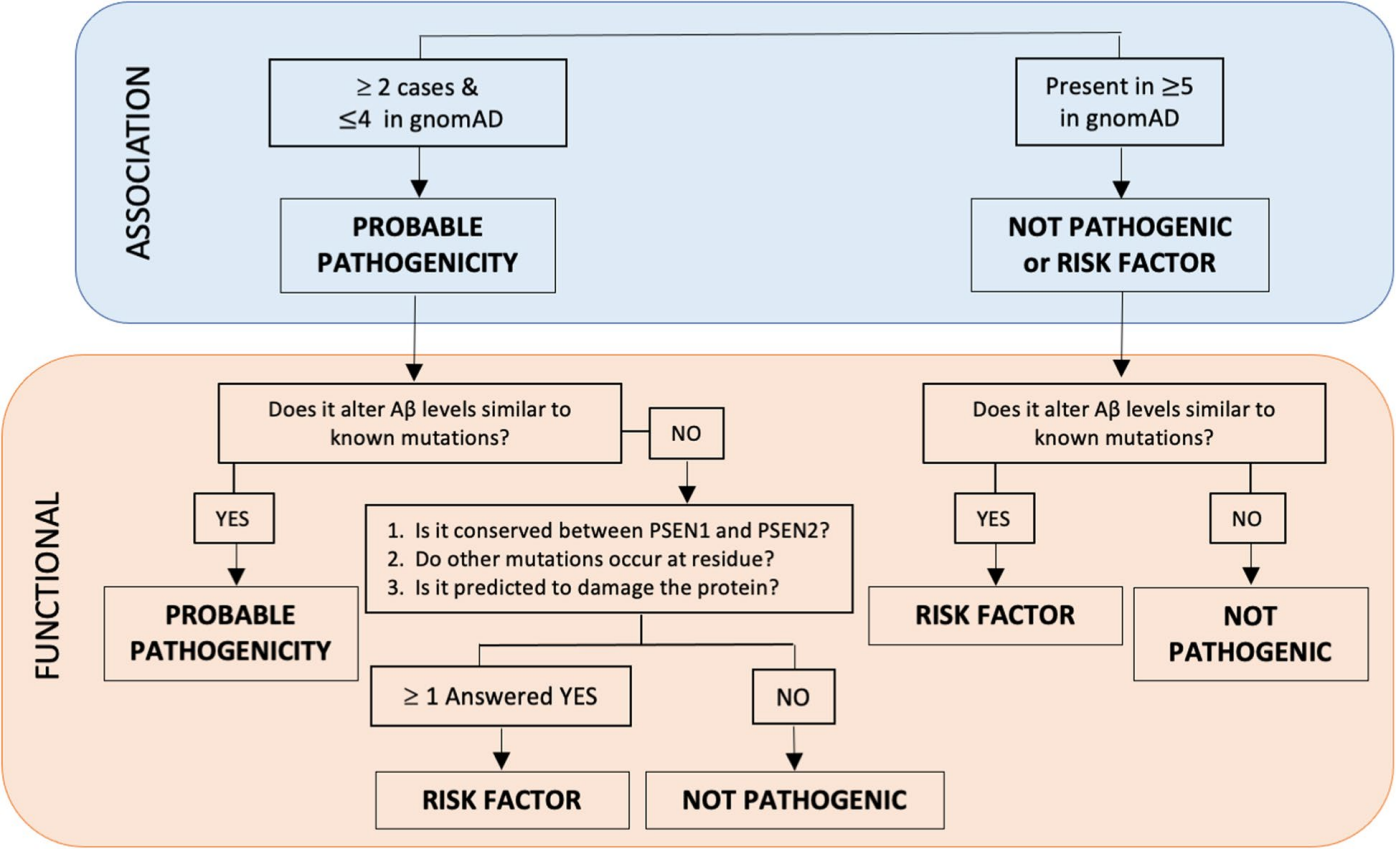
Algorithm to classify the benign or pathogenic nature of DIAD variants. This model is modified from the algorithm previously proposed by Guerreiro et al. in 2010 [23] and Hsu et al. in 2018 [25]

Derived from Guerreiro et al. [23], Hsu et al. proposed a method to integrate bioinformatic and cell-based data when family segregation data is limited or unavailable [24]. First, genetic association is evaluated by determining whether the variant is identified in two or more unrelated AD cases and absent in a large population series. If the variant meets these criteria, the pathogenicity is determined to be probable. Next, functional evaluation weighs the impact of the variant on Aβ levels in a cell-based model. If the variant increases Aβ42, both Aβ42 and Aβ40, or increases the Aβ42/40, it is determined to be likely pathogenic. If the variant does not alter Aβ levels in a cell-based model, conservation is maintained between *PSEN1* and *PSEN2*, other mutations occur at the residue, and in silico analyses predicting damaging effects, then the variant is determined to be a risk factor. If these criteria are not met, the variant is determined to be a benign polymorphism. This approach was used to evaluate the novel variants identified in five kindreds (Fig. 1).

## Results

### Novel variants and clinical features

We identified five novel variants in the *PSEN1* gene (Family A: p.Val103_Ser104delinsGly, Family B: p.Ala275Thr, Family C: p.Lys395Ile, Family D: p.Pro264Se, Family E: p.Ile414Thr). No additional variants were detected in *PSEN2*, *APP*, or other dementia causative genes (e.g., *MAPT*, *GRN*, and *TARDBP*). The main clinical features of the *PSEN1* variant carriers are summarized in Table 1. The mean age at onset in newly identified families was 43.5 years (range 36–54), which is consistent with early-onset AD due to *PSEN1*.

### Family A: p.Val103_Ser104delinsGly

**Fig. 2:**
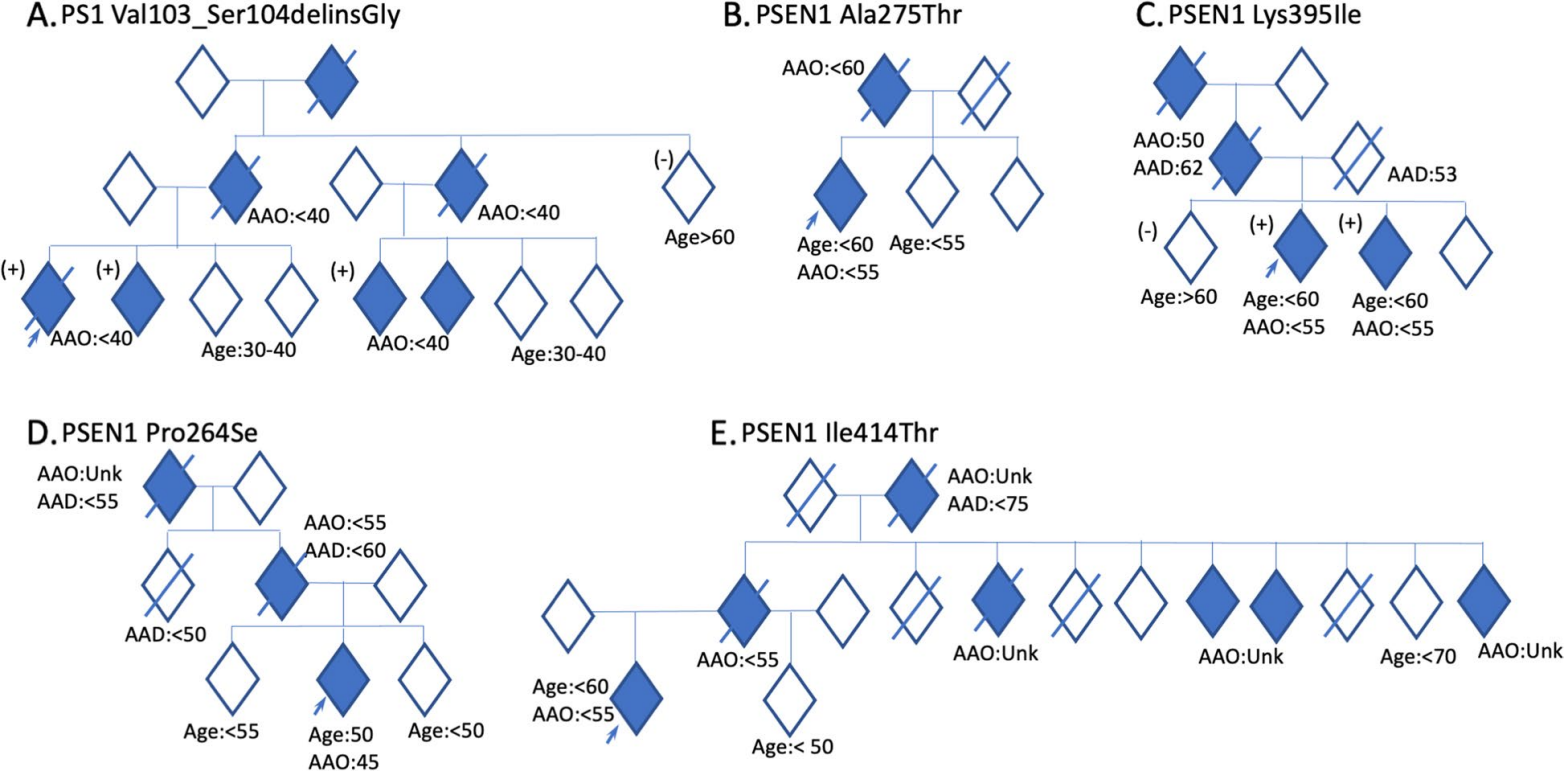
Identification of DIAD variants in densely affected Alzheimer’s disease (AD) pedigrees. Individuals with MCI and dementia have been classified as symptomatic and are represented with shaded rhombus. All generations under the family average age at onset were excluded, and gender has been masked to maintain anonymity. Diagonal lines represent deceased individuals. All symptomatic participants in the study were labeled with symptomatic age at onset. If the age at onset was unknown, the data were labeled as not available (Unk). Arrows indicate the index case. (+) indicate those individuals with DNA, all of whom are mutation/variant carriers. (−) indicate those individuals with DNA, all of whom are NOT mutation/variant carriers. For asymptomatic mutation carriers under the family age, the results of genetic testing were excluded to prevent potential disclosure of mutation status

The proband (patient III1, Fig. 2a) came from a family with a history of early-onset dementia (<65 years) in three generations. Cognitive complaints emerged at age 39 years, characterized by memory loss, word-finding difficulties, and geographic disorientation. The proband was first assessed one year after symptom onset: MMSE score was 19, CDR® was 1, and Functional Activities Questionnaire (Pfeffer) score was 11. Brain MRI showed global atrophy, and FDG-PET revealed hypometabolism in the posterior cingulate cortex, precuneus, and temporoparietal cortices. One year later, the proband started to have myoclonus and the MMSE score was 13. Two years later, the proband was unable to identify family members, got lost inside the house, and could no longer recognize himself in the mirror; MMSE score was eight. *APOE haplotype* was E3/E3. The patient passed away at the age of 44 years due to complications of dementia (pneumonia), 5 years after disease onset. Seizures were described in the last months of his disease. The proband had one sibling diagnosed with early-onset AD (patient III2, Fig. 2a), as well as two first-degree cousins (patient III5 and III6, Fig. 2a).

Sequencing of the proband DNA revealed a *PSEN1* variant that would result in the deletion of the amino acids valine at codon 103 and serine at codon 104 and the insertion of a glycine in place, without changing the translation-reading frame (in-frame) (p.Val103_Ser104delinsGly). The proband’s symptomatic sibling and cousins carry the same variant and the asymptomatic aunt (patient II5, Fig. 2a) and sibling (patient III3, Fig. 2a) did not carry the variant, suggesting that the variant segregated with disease in this family.

**Fig. 3:**
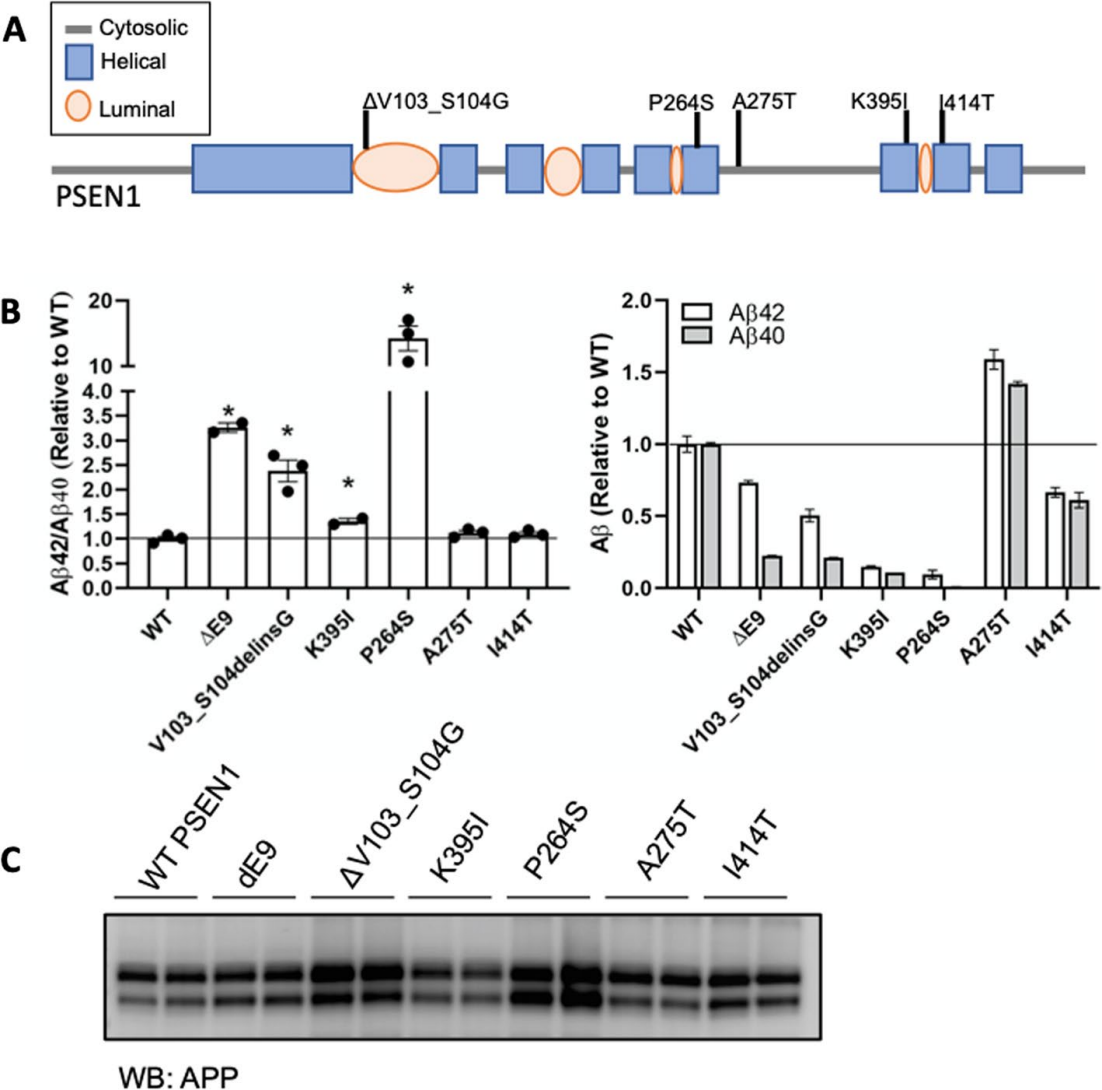
Cell-based model to assess the impact of variants of unknown significance in *PSEN1* on A*β* levels. **A** Diagram of the location of variants of unknown significance in *PSEN1*. **B** Mouse N2A-PS1/PS2 KO cells were transiently transfected with plasmids containing APP WT and *PSEN1* WT, known pathogenic mutation (ΔE9), or a variant of unknown significance. After 48 h, media was collected and analyzed for Aβ42 and Aβ40 by ELISA. B. Ratio of Aβ42/40 expressed relative to *PSEN1* WT. Aβ42 (white box) and Aβ40 (gray box) levels expressed relative to PSEN1 WT. Graphs represent mean ± SEM. Significance indicated by Dunnett’s *t*-test (*, *p* < .05). C. Cells lysates were analyzed by SDS-PAGE and immunoblotting as described in the “Methods” section. Immunoblots were probed with 6E10 (full-length APP). The immunoblot is representative of 2 independent experiments

To explore whether these variants of unknown significance in *PSEN1* impact Aβ levels in a manner consistent with known pathogenic mutations in *PSEN1*, we used a N2A-PS1/PS2 KO cell line in which endogenous presenilin genes are deleted [22, 25]. *PSEN1* p.Val103_Ser104delinsGly produced a significant increase in the extracellular Aβ42/40 ratio compared with *PSEN1* WT, which was consistent with known pathogenic mutations (Fig. 3).

Finally, to determine pathogenicity, we used the algorithm as described by Hsu et al. [24]. *PSEN1* p.Val103_Ser104delinsGly segregates with the disease. This variant was absent from the large population-based gnomAD genome and exome databases (Table 2), with no prior documentation in the medical literature. A missense variant in the same position (p.Val103Gly) [26] was previously reported and identified as a variant of unknown significance. The absence of a rare variant in large, population-based cohorts suggests that the variant may be pathological in nature and thus evolutionarily selected against. This residue is highly conserved between *PSEN1* and *PSEN2*. In a cell model, *PSEN1* p.Val103_Ser104delinsGly, produced Aβ profiles consistent with known pathogenic mutations. Thus, the *PSEN1* p.Val103_Ser104delinsGly variant meets the proposed criteria for designation as pathogenic.

**Table 2:** Variants of unknown significance evaluated by the pathogenicity algorithm

*PSEN1* variant	Variant location	Variant type/consequence	GnomAD ClinVar EVS	SIFT	CADD	Clinical significance according to the ACMG criteria (Varsome)	Clinical significance according to the ACMG criteria (Franklin by Genoox)	In vitro analysis^a^	Clinical significance^b^
p.Val103_Ser104delinsGly	c.308_310del 4 chr14:73637725	Deletion, in frame	NP	NA	NA	Pathogenic	Pathogenic	Yes	AD : Pathogenic
p.Lys395Ile	c.1184A>T 11 chr14:73664760	Point, missense	NP	Damaging	31	Pathogenic	Pathogenic	Yes	AD : Pathogenic
p.Pro264Se	c.790C>T 8 chr14:73683888	Point, missense	NP	Damaging	28.4	Pathogenic	Likely pathogenic	Yes	AD : Pathogenic
p.Ala275Thr	c.823G>A 8 chr14:73664792	Point, missense	NP	Damaging	29.4	Likely pathogenic	Likely pathogenic	Yes	AD : Pathogenic
p. Ile414Thr	c.1241T>C 11 chr14:73683945	Point, missense	NP	Damaging	28.4	Likely pathogenic	Likely pathogenic	No	Not Pathogenic/ Risk factor

*GnomAD* Genome aggregation database (~17720 Latino origen [12.5%]), *EVS* Exome Variant Server, *dbSNP* single nucleotide polymorphisms, *SIFT* Sorting Intolerant From Tolerant, *CADD* Combined Annotation Dependent Depletion, *NP* not present absent as of December 2021

^a^Change in Aβ consistent with pathogenic mutations

^b^Pathogenicity classification based on the algorithm proposed Hsu et al. [25] PSEN1 transcript ENST00000324501.5; reference genome: hg19

### Family B: p.Ala275Thr

The index case (patient II1, Fig. 2b) in this family developed depressive symptoms and loss of episodic memory at 45 years old, followed by aberrant motor symptoms and obsessive-compulsive tendencies, manifesting with increased cleaning behaviors. By 47 years, language problems ensued, and at age 49 (age at which was first assessed) profound expressive aphasia was noticed. There was no history or signs of parkinsonism, myoclonus, psychotic symptoms, or seizures. Brain MRI revealed hippocampi and temporoparietal atrophy. Brain SPECT revealed right posterior parietal hypoperfusion. The proband was diagnosed with early-onset AD. The proband’s parent (patient I1, Fig. 2b) had AD with onset at age 56 years and death at 66 years.

Sequencing of the proband DNA (Fig. 2) revealed a single base pair substitution (GCC to ACC) at codon 275 in exon 8 of *PSEN1*, resulting in an alanine-to-threonine change (p.Ala275Thr). *PSEN1* p.Ala275Thr was absent in from both gnomAD genome and exome databases. This residue was highly conserved between *PSEN1* and *PSEN2*. *PSEN1* p.Ala275Thr has not been reported in ClinVar, but two variants in the same position (p.A275V, p.A275S) were previously reported and identified as pathogenic [27, 28], supporting the damaging effect of amino acid changes in position 275. Cells expressing *PSEN1* pAla275Thr produced a significant increase in A*β*42 and A*β*40 levels without altering the ratio (Fig. 3). These findings suggest that *PSEN1* p.Ala275Thr is a likely pathogenic variant.

### Family C: p.Lys395Ile

The proband (patient III2, Fig. 2c) was identified in a Brazilian family with three generations of early-onset AD and mean age-at-symptomatic onset of 51.3 years. The proband’s symptoms started at the age of 51 years, with episodic memory problems and later development of anomia and geographic disorientation. The proband had no major neuropsychiatric symptoms aside from apathy. Neurological examination revealed a postural tremor of both hands, which had been present since 30s (suggestive of essential tremor) and had mild rigidity in his upper right limb. His first MMSE score (at age 54 years) was 16, with progressive worsening. Four years after the first assessment (seven years after the onset), MMSE score was six. First MRI was interpreted as “normal.” EEG was normal. *APOE haplotype* was E3/E3. Blood tests did not reveal an alternative cause for dementia.

The proband’s parent (patient II1, Fig. 2c) was diagnosed with AD and parkinsonism, with age-at-symptomatic onset of 55 years, and the grandparent (patient I1, Fig. 2b) had similar symptoms with onset at 60 years. The proband’s parent died at age 53 due to a workplace accident. The proband’s sibling (patient III3, Fig. 2a) was diagnosed with early-onset AD with symptomatic onset at age 55 years.

Sequencing of the proband revealed a single base substitution (AAA to ATA) at codon 395 in exon 11 of *PSEN1*, resulting in a replacement of the lysine amino acid at codon 395 by isoleucine (*PSEN1* p.Lys395Ile). The variant was present in several family members, segregating with symptomatic disease. The proband’s sibling carried the same variant (patient III3, Fig. 2c). The asymptomatic older sibling (patient III1, Fig. 2a) did not carry the variant (at the time of testing the older sibling was eight years older than the mean family age-at-symptomatic onset). *PSEN1* p.Lys395Ile was absent from population-based databases (gnomAD genome and exome) and not reported in ClinVar (as of Dec 2021). Lysine at position 395 is highly conserved across species, suggesting that its replacement by isoleucine may be deleterious. In a cell model, *PSEN1* p.Lys395Ile led to a significant increased extracellular Aβ42/40 ratio. Applying the algorithm for assessing pathogenicity, we propose that *PSEN1* p.Lys395Ile represents a pathogenic variant.

### Family D: p.Pro264Ser

The proband (patient III2, Fig. 2d) was identified in a family with three generations of early-onset AD and with a mean age-at-symptomatic onset of 48.5 years. The proband started forgetting messages at work when she was 45 years old. Memory problems worsened over the coming 1–2 years, eventually requiring cessation of work. The proband had episodes of geographic disorientation and became more disorganized. During the first clinical assessment (at age 48 years), the MoCA score was 22 (points lost for impaired clock draw and inability to recall five words after a delay). Neuropsychological testing confirmed impaired verbal and visual recall, with relative preservation of semantic and phonemic fluency.

Sequencing of the proband (patient III2, Fig. 2d) revealed a single base pair substitution (CCT to TCT) at codon 264 in exon 8 of *PSEN1*, resulting in a proline-to-serine change (*PSEN1* p.Pro264Ser). This variant was not identified in ClinVar (as of Dec 2021) and was absent from gnomAD genome and exome databases (Table 1). This residue is highly conserved between *PSEN1* and *PSEN2*. Another variant in the same position (p.Pro264Leu) [29, 30] was previously reported and identified as pathogenic, supporting the damaging effect of amino acid change in position 264. Cells expressing *PSEN1* p.Pro264Ser produced a significant increase in the extracellular A*β*42/40 ratio (Fig. 3). Thus, applying the algorithm for assessing pathogenicity, we propose that the *PSEN1* p.Pro264Ser represents a likely pathogenic variant.

### Family E: p.Ile414Thr

The proband (patient III1, Fig. 2e) developed irritability and apathy at 51 years old, followed by progressive memory loss. At baseline assessment, the proband scored 23/30 on the MMSE. Global CDR® was 0.5 (very mild dementia). Reassessment one-and-a-half years later revealed rapid progression in the clinical course, scoring 16/30 on the MMSE. Global CDR® was 2 (moderate-severity dementia). Ideomotor apraxia was present on examination. No myoclonic jerks or abnormal movements were observed. APOE haplotype was E3/E4. The proband’s parent developed a “progressive memory decline” at age 50, and several family members (Fig. 2) showed similar clinical presentation. This family originated in a remote community in rural Mexico, with a few family members dispersed across Mexico City. To date, 6 of 12 family members within the age at onset range have reported cognitive impairment, three of whom are deceased. Segregation of the variant was not possible in this family. The mean age-at-symptomatic onset in this family is 59.0 years (range 50–74).

Sequencing of the proband (patient III1, Fig. 2e) revealed a single base pair substitution (ATT to ACT) at codon 414 in exon 11 of PSEN1, resulting in an isoleucine-to-threonine change (*PSEN1* p.Ile414Thr). *PSEN1* p.Ile414Thr was absent in 250 Mexican mestizo exomes and from both gnomAD genome and exome, and has not been reported in ClinVar (as of December 2021). This residue is highly conserved between *PSEN1* and *PSEN2*. Cells expressing *PSEN1* p.Ile414Thr produced Aβ42 and Aβ40 levels similar to *PSEN1* WT and did not alter Aβ in a manner consistent with a known pathogenic mutation. While the bioinformatic predictions are consistent with potential pathogenicity, bioinformatic findings alone are not sufficient to define pathogenicity. Based on the current evidence (insufficient segregation data in the affected family and in vitro results indicating that *PSEN1* p.Ile414Thr does not alter Aβ), we propose that *PSEN1* p.Ile414Thr represents an AD risk factor.

Finally, for all the novel variants, N2A PS1/PS2 KO cells were co-transfected with APP and PSEN1 variants to further analyzed for APP expression. Expression was confirmed with immunoblotting, suggesting the alteration in Aβ42/40 levels in PSEN1 variants was independent of APP expression (Fig. 3C).

## Discussion

We identified one novel frameshift deletion in *PSEN1* and four novel missense variants in *PSEN1* in LatAm families from Brazil and Mexico. These novel variants were not present in the public sequencing databases including population-matched individuals or population-matched exomes (as of Dec 2021). Four of these variants (p.Val103_Ser104delinsGly, p.Lys395Ile, p.Pro264Se, p.Ala275Thr) showed evidence of elevated levels of Aβ42 and Aβ42/Aβ40 ratios, similar to known pathogenic *PSEN1* variants and were classified as likely pathogenic. Additionally, two *PSEN1* variants (p.Val103_Ser104delinsGly and p.Lys395Ile) were shown to segregate with disease in each family, which is the gold standard for pathogenicity. We did not have sufficient evidence to classify *PSEN1* p.Ile414Thr as pathogenic. According to the ACMG/CAP guidelines [31], *PSEN1* p.Ile414Thr would have been classified as likely pathogenic. The position Ile414 and nucleotide c.1241 are highly conserved across species and the amino-acid change is predicted to be deleterious using *in silico* programs (BayesDel_addAF, DANN, DEOGEN2, EIGEN, FATHMM-MKL, LIST-S2, M-CAP, MVP, MutationAssessor, MutationTaster, PrimateAI, and SIFT). The variant is absent from the population databases, including 250 Mexican Mestizo individuals supporting that it is not a common variant in the Mexican population. However, in vitro assay results showed this variant did not alter Aβ levels in a manner consistent with pathogenic mutations, and segregation studies were not possible for this family. Future studies should explore this variant in more detail.

Pathogenic presenilin mutations are enriched in the transmembrane domain. Interestingly, the variants of unknown significance were also enriched in the transmembrane domain and are highly conserved between PSEN1 and PSEN2. The location and conservation of these variants support a prediction of likely pathogenicity based on in silico analyses alone [23].

PSEN1 p.Val103_Ser104delinsGly, p.Pro264Ser, and p.Ala275Thr have not been previously reported. However, these sites have other mutations reported: p.V103G, p.Pro264Leu, p.Ala275Ser, and p.Ala275Val. PSEN1 p.V103G and p.Ala275Ser have a clinical presentation of AD but the pathogenicity has not yet been confirmed, whereas PSEN1 p.Pro264Leu and p.Ala275Val have been classified as pathogenic and likely pathogenic, respectively [28, 29, 32–34]. Pathogenic mutations have not been described at sites impacted by PSEN1 p.Lys395Ile and p.Ile414Thr. Notably, PSEN1 p.Pro264Ser and p.Lys395Ile occur near the aspartate residues that regulate the catalytic activity of PSEN1. Pathogenic mutations PSEN1 p.Pro267Ser and p.Pr0355Ser also occur near the aspartate residues and have been shown to alter γ-secretase activity [35–37]. Thus, these variants of unknown significance may impact the Aβ42/Aβ40 ratio via disrupted catalytic activity of PSEN1 and the γ-secretase complex. PSEN1 p.Ala396Thr has previously been reported to lead to increased Aβ40 and Aβ42 production without a change in the Aβ42/Aβ40 ratio in mouse neuroblastoma cells, while an independent cell-free assay suggests that PSEN1 p.Ala396Thr leads to reduced Aβ40 yielding an increase in Aβ42/Aβ40; thus, which the mechanism of the effect remains to be resolved the disruption in Aβ processing is consistent [21, 25, 38].

*PSEN1* pathogenic variant carriers had a variable clinical presentation, including memory impairment, behavioral changes, language, and extrapyramidal symptoms consistent with what we know about PSEN1 mutations. Our study found differences in age-at-symptomatic onset (range, 38–55 years), rate of progression, and clinical presentation for each variant and within families (Table 2). Heterogeneity in age-at-symptomatic onset and clinical presentation in DIAD populations has been highlighted in previous studies [39, 40]. Differences in the mutation (missense vs. in-frame splice) or location (codon <200 vs.>200) may impact the pathologic function of PSEN1, contributing to differences in biochemica l[41] and phenotypic expression of disease. However, as suggested by Ryan et al., family mutation alone does not explain the large proportion of the observed heterogeneity [39, 40, 42, 43], suggesting that other environmental or genetic factors modify the effect of *PSEN1* mutations.

Several known DIAD variants from Latin America show relevant evidence of geographical cluster and funders’ effects. We could not identify such evidence in the novel variants described in this study, suggesting a possible de novo origin in these families.

Our findings are limited by (1) the absence of AD biomarkers and (2) the lack of large genetic databases with sequencing data on LatAm populations.

Due to limitations in local resources, AD biomarkers were not collected in any of the family members. The newly developed blood-based biomarker (e.g., plasma Aβ42 and Aβ42/Aβ40 ratios and plasma phosphorylated tau proteins [ptau181, ptau217, ptau205]) [44–46] may represent an alternative for future studies and is especially relevant for underserved communities. The development and activation of new DIAN sites in LatAm (Argentina, Colombia, Brazil, Mexico) will facilitate future biomarkers collection in this population in DIAD families from the region. Newly identified families have been invited to join ongoing observational studies and clinical trials in DIAN sites.

To determine whether the novel variants represented rare or common polymorphisms, we examined population-based sequencing databases. This analysis is limited by the lack of diverse representation in these databases that are largely populated by individuals of European-American descent [47], raising the possibility that these variants could be seen with greater frequency in African or Native American populations which would reduce their likelihood of being pathogenic. Population stratification is particularly important when considering rare variants. It is important to highlight that genetic testing and counseling in this study was restricted to families with two or more generations with a history of early-onset dementia. Large population-based and more inclusive studies aimed at identifying novel genetic variants in Latin America remain critical but unfortunately limited by funding. Finally, although genetic counseling was offered to family members, attempts to maintain confidentiality raised specific challenges to document family history and discuss further genetic testing among the family; therefore, segregation was not available for all the families.

Our study provides further insights into the genetics of AD in LatAm by identifying five novel mutations in the *PSEN1* gene. This report is a step forward to improving the inclusion/engagement of LatAm families in research. Genetic counseling and testing are now available for at-risk, cognitively healthy individuals who may opt to learn their genetic status, decide about reproductive choices, and enroll in clinical trials. Family discovery is of great relevance for the region, as new initiatives are underway to extend clinical trials and observational studies to families living with DIAD. Broader inclusion of families with DIAD from across the world is likely to increase our understanding of the pathogenic variants that contribute to AD, with the potential to inform determinants and modifiers of AD pathogenicity, improve the care of patients and families with DIAD, and advance the development and evaluation of putative AD-modifying therapeutics.

## Conclusions

Based on our findings, including clinical presentation, imaging, genetic, segregations studies, and cell-based analysis, we propose that *PSEN1* p.Val103_Ser104delinsGly, p.Lys395Ile, p.Pro264Se, and p.Ala275Thr are likely pathogenic variants resulting in DIAD, whereas *PSEN1* p.Ile414Thr is likely a risk factor.

## Data Availability

The datasets generated during and/or analyzed during the current study are not publicly available due to participant confidentiality but are available from the corresponding author on reasonable request.
